# Development of a novel anti‐amyloid therapeutic: an orally administrable anti‐abeta‐oligomer compound in early preventative intervention

**DOI:** 10.1002/alz70859_101967

**Published:** 2025-12-25

**Authors:** Hedi Zhou, Adeola Shobo, Mark Hancock, Fatima El Ansari, Sara Touj, Medhinee Malvankar, Pei You Wu, Louis‐Charles Masson, Thierry Choquette, Mallar M. Chakravarty, Rebecca Anne McKinney, Gerhard Multhaup

**Affiliations:** ^1^ McGill University, Montreal, QC Canada; ^2^ Douglas Mental Health University Institute, Montreal, QC Canada; ^3^ Cerebral Imaging Centre, Douglas Mental Health Institute Research Centre, Montreal, QC Canada

## Abstract

**Background:**

Over the past decade, the Multhaup lab have evaluated the safety and efficacy of a protease resistant D‐amino acid Aβ‐Interacting Peptide (D‐AIP) as a novel anti‐amyloid preventive strategy, which targets Aβ accumulation as the primary event in Alzheimer disease (AD) pathogenesis. D‐AIP selectively binds to soluble oligomers of Aβ42 in vitro (Barucker et al. 2015), neutralizes Aβ42 oligomer toxicity in *Drospohila* models (Zhong et al. 2019), and crosses the blood‐brain barrier in wild‐type mice (Shobo et al. 2022). Here, we investigated effects of D‐AIP on early Aβ pathogenesis in 3xTg‐AD mice.

**Method:**

3xTg mice were orally treated with D‐AIP from 4 to 6‐months‐old. Liquid chromatography mass spectrometry was used to detect and quantify D‐AIP in brain homogenates and plasma. D‐AIP and Aβ42 oligomers were localized in 3xTg brains by matrix‐assisted laser desorption ionization mass spectrometry imaging (MALDI‐MSI). Immunohistochemistry was used to track amyloid pathology, microglia and astrocyte reactivity in brain sections. An ultrasensitive Meso Scale Discovery immunoassay was used to quantify Aβ species and follow the progression of amyloid deposition.

**Result:**

Orally dosed D‐AIP possessed favourable biostability, pharmacokinetics, and brain region distribution. Notably, (i) D‐AIP forms heteromeric complexes with toxic Aβ oligomers in the brains of 3xTg‐AD animals, (ii) attenuated plaque amyloid pathology and (iii) attenuated neuroinflammation at the lag‐phase of amyloid aggregation in male and female 3xTg mice. Additionally, behaviour and structural analysis showed that D‐AIP treatment had no adverse effects on memory and cognition.

**Conclusion:**

Our findings demonstrate that oral administrated D‐AIP effectively targeted Aβ oligomers to prevent AD‐associated deposition and neurotoxicity in an AD mouse model at very early stages of amyloid deposition. Since orally delivered D‐AIP had no observable adverse effect it presents great promise as a next‐generation AD therapeutic.

